# Animal-Based Indicators for On-Farm Welfare Assessment in Goats

**DOI:** 10.3390/ani11113138

**Published:** 2021-11-02

**Authors:** Adrian Minnig, Romane Zufferey, Beat Thomann, Sibylle Zwygart, Nina Keil, Gertraud Schüpbach-Regula, Raymond Miserez, Dimitri Stucki, Patrik Zanolari

**Affiliations:** 1 Clinic for Ruminants, Vetsuisse-Faculty, University of Bern, 3012 Bern, Switzerland; adrian.minnig@students.unibe.ch (A.M.); romane.zufferey@students.unibe.ch (R.Z.); patrik.zanolari@vetsuisse.unibe.ch (P.Z.); 2 Veterinary Public Health Institute, Vetsuisse-Faculty, University of Bern, 3012 Bern, Switzerland; beat.thomann@vetsuisse.unibe.ch (B.T.); gertraud.schuepbach@vetsuisse.unibe.ch (G.S.-R.); 3 Centre for Proper Housing of Ruminants and Pigs, Federal Food Safety and Veterinary Office, Agroscope, 8356 Tänikon, Switzerland; sibylle.zwygart@vetsuisse.unibe.ch (S.Z.); nina.keil@agroscope.admin.ch (N.K.); 4 Consulting and Health Service for Small Ruminants, 3362 Niederönz, Switzerland; raymond.miserez@caprovis.ch

**Keywords:** goat, small ruminant, animal-based, welfare assessment, welfare indicators, on-farm

## Abstract

**Simple Summary:**

Welfare assessments for animals require the use of specific indicators. These indicators should be practical and easy to use in an on-farm environment while correctly reflecting on the animals’ welfare. Our aim was to review literature on such indicators for goats, as small ruminants have not received as much attention as other farm animals in the field of welfare assessment. Some indicators such as lameness are already well investigated and suitable for use in goat welfare assessments. Others, for example, lying behaviour, need more research, as the limited amount of knowledge restrains the information on validity or usefulness. As in other animals, the welfare of goats has become an increasingly important issue in public discourse. Our overview on indicators aids in developing tools to measure and improve the welfare of goats.

**Abstract:**

This review describes the current state of knowledge relating to scientific literature on welfare indicators for goats. Our aim was to provide an overview of animal-based indicators for on-farm welfare assessments. We performed a literature search and extracted 96 relevant articles by title, abstract, and full-text screening. Out of these articles, similar indicators were aggregated to result in a total of 32 welfare indicators, some of which were covered in multiple articles, others in only a single one. We discuss a set of three established assessment protocols containing these indicators, as well as all individual indicators which were covered in more than one article. As single indicators, we identified lameness, body condition score (BCS), qualitative behaviour assessment (QBA), and human–animal relationship (HAR) tests with substantial evidence for sufficient validity to assess welfare in goats. A multitude of indicators (e.g., hair coat condition) was studied less intensively but was successfully used for welfare assessments. For some indicators (e.g., oblivion, lying behaviour), we highlight the need for future research to further validate them or to optimise their use in on-farm welfare assessments. Moreover, further investigations need to include kids, bucks, and meat and fibre goats, as well as extensively kept goats as the literature predominantly focuses on dairy goats in intensive production systems.

## 1. Introduction

Animal welfare encompasses the quality of living for animals which is measurable at a particular time [1]. Consumer demand for certified animal welfare has been increasing during the last few decades, leading to a gain in importance for welfare assessments in animal husbandry [2]. Small ruminants have not received as much attention as other farm animals regarding possible ways to assess welfare. However, with the rising number of larger goat herds in more intensive production systems, welfare issues have increased [3]. The starting point for on-farm welfare assessment is the selection of suitable animal-based and/or resource-based indicators [3]. It is argued that animal-based indicators may be more appropriate to measure the animals’ actual welfare because environmental (resource-based) aspects may show high variation depending on housing and management conditions [2].

A valid indicator can accurately measure welfare and correlates to an existing validated indicator or to a conceptually related measure [4]. Reliability of an indicator includes its ability to produce similar results when tested by multiple different assessors (inter-observer reliability) or by the same assessor on multiple occasions (intra-observer reliability) [4]. Consistency over time is another aspect of reliability and describes the consistency of results from the same test, performed at two different times [4]. Feasibility includes practical aspects of using indicators on-farm, time- and cost constraints, and acceptance of indicators by farmers and stakeholders [2].

In the last decade, research on goat welfare has made significant progress with the development and testing of assessment protocols in the United Kingdom [5], in Norway [6], in Portugal and Italy [7], and in North America [8,9]. However, most research, including these protocols, mainly focus on dairy goats kept in intensive production systems. Little information is available on the welfare of bucks, kids, meat- or fibre goats, or goats kept for extensive periods.

In this review, we describe the current state of on-farm welfare assessment in goats. We identified possible animal-based indicators and discuss their ability to reflect on goats’ welfare. Unlike previous reviews of goat welfare, for example, [2], our review is not focused on dairy goats specifically. We provide a diverse selection of indicators that could be applied in different production systems. In addition, in contrast to summarising the available indicators, we specifically extracted available information on the respective study authors’ judgment of the indicators’ quality with respect to validity, reliability, repeatability, and feasibility. For the selection of appropriate animal-based welfare indicators, we focused mainly on the two criteria of validity and reliability, and where available, we also provide available information on feasibility.

## 2. Materials and Methods

### 2.1. Literature Search

We used a series of predefined search terms to find relevant literature for this review. The terms “goat”, “small ruminant”, “goat AND kid”, and “goat lamb” were each combined with 12 other general terms covering the categories of data collection, welfare, health, and mortality. We searched the four literature databases PubMed [10], Web of Science [11], Scopus [12], and ScienceDirect [13] using these combined terms between 23 February 2020 and 7 March 2020. The language setting was restricted to articles written in English, German, or French. Articles written before 1 January 1995 were excluded. When a particular term combination returned more than 500 results, we applied website-specific filters such as “Veterinary Sciences” or “Species: animals” to limit results to a reasonable number. In total, we found 12,413 results and imported these into the literature management program Zotero [14]. The number of results and procedure (terms, filter application, dates) was documented in a MS Excel [15] spreadsheet.

### 2.2. Title and Abstract Screening

We uploaded all articles to DistillerSR^®^ [16], a tool for performing systematic reviews. We used this tool to perform a duplicate check which excluded approximately 4958 results. The remaining 7455 articles were then subjected to title screening. We considered only peer-reviewed original articles or reviews focusing on indicators for goats. We excluded publication titles not considered relevant and chose 450 articles for abstract screening. From these results, we chose 81 abstracts for review and categorisation. One article was withdrawn in April 2020 because of plagiarism. We checked the original article, which was plagiarised, and decided that it was not relevant for this review, leaving a total of 80 articles for data extraction.

### 2.3. Full-Text Screening and Data Extraction

From the set of articles for data extraction, we identified animal-based indicators or, if covered by the article, entire assessment protocols. By reviewing the references of these 80 articles, we identified an additional 16 articles as potentially relevant, which usually did not feature our search terms in their title or abstract. The identified indicators from the resulting 96 articles were listed and documented in MS Word [17] and MS Excel [15]. Five articles described procedures that included multiple indicators to create an animal welfare assessment protocol. Including the indicators used in these protocols, we identified a total of 32 individual indicators (indicators with similar definitions were grouped). In the following, we first describe the five protocols as an overview of the available methods to assess goat welfare as a whole. We then discuss each identified indicator in sequence of the number of articles addressing the indicator. Where the respective information is available, we discuss the indicators with respect to the reported validity (does the indicator reflect welfare?), reliability (how accurate is the indicator?), and feasibility (how easy is the indicator to apply?).

## 3. Results and Discussion

In the following section, we describe how welfare assessment protocols and individual welfare indicators are used in the field and we discuss their advantages and disadvantages. We first specifically present the four articles that provide entire assessment schemes in the form of protocols. The protocols are presented in order of the extensiveness of on-farm testing, beginning with the most validated protocol. The indicators featured in each protocol are shown in Table 1, providing an overview of the frequency of use of the separate indicators among the protocols. Second, we present and discuss all individual articles we identified from the available literature. Individual indicators are presented in order of the number of articles that feature the indicator, commencing with the indicators with many supporting articles. Only indicators that are featured in more than three articles are discussed in detail, whereas indicators with only little information in the literature are listed in Table 2.

### 3.1. Welfare Assessment Protocols for Goats

#### 3.1.1. AWIN Protocol

The Animal Welfare Indicator (AWIN) project [18] was created to develop animal-based protocols for assessing the welfare in different animal species, including adult dairy goats in intensive or semi-intensive (occasional access to pasture) production systems. The selection of possible indicators for a prototype protocol was based on the “Principles and Criteria of Good Welfare” defined by the Welfare Quality^®^ of the European Union [19]. Validity, reliability, and on-farm feasibility were used by Reference [2] as criteria for indicators of the prototype protocol. After testing the prototype protocol on a total of 60 intensive dairy goat farms in Italy [20] and Portugal [21], we found that 18 indicators remained for the final protocol. Four of the indicators were additionally validated in more detail by researchers involved in the AWIN projects, namely, hair coat condition [22], thermal stress [23], body condition score [24], and qualitative behaviour assessment [25]. These indicators will be discussed in detail below in Section 3.2: Individual Welfare Indicators for Goats.

The protocol is applied on-farm in two separate phases. Phase one consists of a general herd investigation for a set of indicators, which are quick and easy to assess. Only if welfare violations are encountered or if the farm ranks among the 5% lowest scores for any indicator is phase two performed. In phase two, an assessment of a slightly different set of indicators is applied on an individual animal level. This two-phase approach vastly increases assessment times for farms with little concern. Results from prototype-testing in Northern Italy [20] showed an average total assessment time of 140 ± 8 min. Reference [21] presents a mean overall time of 87 ± 33 min required to apply the protocol in Portuguese farms. Compared to the duration of other goat welfare assessment protocols, reported as “one working day” in Reference [6] and from one morning to milking at the following morning in Reference [5], the AWIN protocol appears to be more feasible considering time constraints. However, it is important to keep in mind that Reference [6] also evaluated resource-based indicators and that Reference [5] acknowledges that most of their measures needed further development in terms of feasibility. Another difference that affects the time required is the number of assessors. While References [5,6] both tested with only one assessor, single animal assessment in the AWIN prototype-test was conducted by two assessors. The focus on very quick assessment of welfare and high feasibility in general is a specific attribute of the AWIN protocol. Assessment techniques such as rating scales were often simplified compared to methods used in other studies. This process increased feasibility on-farm but often reduced measurement accuracy.

Consistency over time is crucial for assessing welfare [2] to indicate the representativeness of results across long-term situations [26]. During prototype testing of the AWIN protocol, out of the 30 visited farms in Portugal and Italy, 10 farms per country were selected to test for consistency over a time period of four months [26]. Over the four-month interval, of all tested indicators, only body abscesses and cleanliness of the lower leg were consistent as defined by the authors. The authors cited several causes for variation that can lead to low consistency, including differences in prevalence; changes in management, feeding, or lactation stages; a disease outbreak; and seasonal effects. Reference [26] indicates that there were no major changes in management or housing on the farms visited but suggested that slight changes in management routines may have caused variability of some indicators. This led the authors to suggest that some indicators might be too sensitive, causing higher variation. Sample size and sampling-strategy, as well as intra-observer effects, were cited as limitations of this work as they could lower consistency over time as well. Finally, the authors noted that two visits are likely insufficient to accurately represent variability for a time period longer than the four months in their study.

Inter-observer reliability of the AWIN protocol was tested on 0 farms each in Northern Italy and Portugal [27]. This aspect of reliability is important for the evaluation of indicators as high levels of inter-observer reliability promote their use in the assessment of welfare, as shown in work on sheep [28,29,30,31]. Two pairs of observers, one pair on 10 farms in Italy and one pair on 10 farms in Portugal, applied the AWIN prototype protocol. Results showed “substantial” inter-observer reliability values for most indicators on the group level (e.g., hair coat condition or queuing at feeding). Individual-level assessment generally showed lower inter-observer reliability values but for most indicators, such as udder asymmetry or overgrown claws, and values were still between “fair to good” and “excellent”. The authors explained this difference between the two levels of assessment by the fact that the time for assessing individual animals was too short for the large number of indicators. Another difference was that data collected by observers in Portugal generally showed better results for inter-observer reliability at the individual-level than results presented by their colleagues in Italy. According to Reference [27], this was mainly caused by the fact that observers in Portugal manually restrained the animals in almost all farms, while in Italy, the animals in four farms were restrained in the feeding rack without manual restriction. Other reasons proposed by the authors for the differences found between countries were the different background and experience of observers, and the prevalence of indicators [32]. Compared to similar studies such as Reference [33], fewer observers per farm (two) were used for the assessment. The authors argued that this limitation was overcome by collecting data from two different countries.

To our knowledge, the AWIN protocol for adult dairy goats is the most comprehensive animal-based protocol for assessing goat welfare in terms of research and testing that has been conducted thus far. The non-invasive nature and short duration increased on-farm feasibility and the stakeholder’s agreement [2]. However, as noted by the authors, part of the reason for high acceptance is that stakeholders were consulted during the development of the protocol. Due to the design, the study is not applicable to extensively kept goats, non-lactating goats, bucks, and kids, and extrapolation of methodologies is limited by the heterogenous nature of extensive environments [34]. With regard to non-lactating goats, Reference [18] notes that many indicators could also be relevant for these animals but specific studies are lacking. Few possible indicators identified by Reference [2] have been validated for goat kids or bucks, but the authors present the AWIN protocol as a basis for developing protocols specifically targeting these categories. Another goal defined by the participating researchers was to increase the size of their reference population and to update their data. In this way, further refinement will be possible to overcome the current limitation that the results are based only on 60 farms in two countries.

#### 3.1.2. Anzuino Protocol

Before the development of the AWIN project, an assessment protocol comparable to the AWIN project had been developed and tested in 2004 and 2005 by Reference [5]. The authors designed their protocol for adult dairy goats and chose direct animal-based observations that minimally disrupted farm routines (Table 2). To validate the protocol by Reference [5], a single observer visited 24 commercial dairy goat farms in England and Wales. During farm recruitment, the authors acknowledge a bias towards the selection of larger farms as the 24 farms represented 37% of the total goat population in the United Kingdom with a median herd size of 496 adult female goats. In terms of feasibility, the authors noted that measures were considered practical to use on-farm (no specific tools or further laboratory work are required for evaluation). Yet, the assessment methods need further development. As mentioned earlier, the application of the protocol is time-consuming (roughly 24 h) and requires a milking parlour to conduct observations of individual animals. Because observations were only carried out by one observer, no information on inter-observer reliability is available. However, since the indicators strongly resemble those of the AWIN protocol, similar inter-observer reliability can be assumed. Nevertheless, the single observer evaluated their intra-observer reliability (repeatability) on a large commercial farm before visiting the 24 farms selected for validating the welfare assessment. The authors of Reference [5] describe their work as an initial study with sparse evidence supporting their assessment parameters, especially in comparison to other species. Nevertheless, as many of the indicators selected for the AWIN project directly originate from the information provided by Reference [5], the Anzuino protocol demonstrated a high impact on past research on goat welfare. The study also recognised the need for research on further behaviours, such as fearfulness, and its effect on welfare.

#### 3.1.3. Muri Protocol

A welfare assessment protocol that combined animal-based and management-based indicators was developed and tested by Reference [6] in Norway. The authors reviewed relevant literature on goat health and welfare as well as welfare assessment in other animal species to produce a first draft, which they then reduced to a final set of indicators (Table 1). Preliminary tests of the feasibility and relevance of indicators were conducted by three observers on “several” goat farms (the precise number is not specified). Regarding reliability, inter-observer agreement was calculated and used to modify the scores and their cut-offs at the individual animal level. For some variables, the data could not be used for statistical analysis due to their “homogeneity” (low variation in prevalence). Reference [35] reports facing similar issues with “homogeneity” when assessing equine welfare. Other indicators showed moderate to near perfect inter-observer reliability. The authors acknowledged the limitations of their reliability data and stated that assessment is needed at the farm-level, not only at the individual animal level, as suggested by Reference [36] for cattle. Reference [6] suggests evaluating the inter- and intra-observer reliability of their final protocol more thoroughly with larger sample sizes as their work was only a calibration for the final modifications.

Farm recruitment was based on several factors, including geographic proximity to other farms, disease eradication status, and farmer availability. The number of farms selected was limited to 30 due to financial reasons. Although these selection criteria may result in some representation bias, the authors still attribute some external validity to their results for the national goat population of Norway. Each of the three observers visited 10 farms and collected data within one working day. As mentioned above, the authors recognised the need to collect data in a shorter time than “one working day” and suggested that in the future, the assessment should be performed by agricultural extension services or by the authorities to reduce time and increase feasibility in this way. Using the protocol, observers first assessed behaviour at the group level and then individually assessed 20 randomly selected goats per herd. One advantage this protocol shares with the AWIN protocol is that individual animal observation can be conducted with goats restrained in the feeding rack and, therefore, a milking parlour is not necessarily required.

Despite the previously mentioned limitations in terms of feasibility and reliability, the authors presented a comprehensive protocol for identifying welfare problems in dairy goat farms. They concluded that their experiences could be useful in developing other welfare assessment protocols for goats. The fact that the average herd size for testing was generally smaller compared to protocols in References [5,7], which may be of interest for the application of welfare assessment protocols in other countries with similarly small goat herds.

#### 3.1.4. Leite Protocol

Reference [37] constitutes a selection of animal- and resource-based indicators for the welfare of meat goats in Brazil (Table 1). The authors selected indicators that were already used in the AWIN protocol for dairy goats [18] and for sheep [38] and added the parameter “cleanliness of facilities”. The indicators were chosen to represent conditions in North Eastern Brazil, where extensive rearing is the predominant production system. Goats in this region experience few management practices and feed mainly on pasture. The authors noted that the proposed indicators could also be applied in semi-intensive and intensive systems. The authors of Reference [37] justify the selection of indicators validated for the AWIN sheep protocol on the basis that, unlike the protocol for dairy goats, it was designed for different rearing systems, including more extensive ones. It is important to note that the authors have not yet tested their protocol on-farm and therefore emphasised the need of assessing the indicator’s validity, reliability, and feasibility for meat goats. Reference [37] also mentions the possibility of evaluating goat bucks’ welfare with the proposed indicators, but further work is needed on this topic as well. In summary, the authors present a set of indicators for assessing mostly extensively raised meat goats, an area that has received little attention from goat welfare research. However, for a definitive verdict, more research is needed to determine if this approach is valid, reliable, and feasible.

### 3.2. Individual Welfare Indicators for Goats

#### 3.2.1. Lameness

In the literature, lameness is described as one of the most serious health and welfare problems for dairy cattle [39,40]. Information for goats, however, is scarce and based on rather small samples. For example, Reference [41] shows decreased milk yield in lame goats, and Reference [42] presents longer kidding intervals for goats with lameness. The inclusion of gait evaluation in the assessment of goat welfare is very common, but there is considerable variation in how researchers evaluate and interpret this parameter. The AWIN protocol is limited to the assessment of severe lameness. The authors state that a standard protocol for identifying mild, moderate, and severe cases would not be feasible on-farm due to the diversity of management practices, used resources, and husbandry constraints on dairy goat farms. As described in Reference [7], goats are assessed at the group and individual levels in the pen. During this assessment, the observer looks for abnormal gait, head nodding, spine curvature, and appearance of kneeling in locations other than the feeding rack as indications of severely lame animals. This method offers good on-farm feasibility with the ability to quickly identify severe cases of lameness. When testing the AWIN prototype protocols, References [20,21] report prevalence of severe lameness of 2.1% and 3.1%, respectively. References [26,27] found a similar prevalence of severe lameness of 1.2–2.5%, and Reference [5] found a similar frequency of severely lame goats in U.K. herds at 3.2%.

Regarding reliability, Reference [27] found considerable inter-observer reliability for the “severe lameness” indicator on the basis of data collected in 10 Italian farms. Decent short-term intra-observer reliability was also found by Reference [41] when describing lameness in a dairy goat herd in Greece. “Severe lameness” was described by Reference [26] with results trending towards significance between two visits. Therefore, consistency over time was judged high enough to be included in the final AWIN protocol. However, according to Reference [21], one obvious limitation of the AWIN protocol is the lack of information on mild and moderate cases.

Reference [5] evaluated lameness in U.K. dairy goats using a four-point scale to assess animals leaving the milking parlour and in their pens. Assessing goats individually in the milking parlour was time-consuming, reducing feasibility. By contrast, assessing the animals in the milking parlour could be beneficial in terms of sensitivity because only assessing goats in pens interspersed with soft bedding such as straw is likely to result in an underestimation of lameness [5]. This was underscored by the fact that the authors found that prevalence recorded in pens was generally much lower than when observed with goats leaving the parlour. Reference [43] provides comparable data on British dairy goats examined while walking on straw in their pens as he noted a prevalence of only 9.1% lame goats compared to 19.2% presented in Reference [5].

In their evaluation of the welfare of Norwegian dairy goats, Reference [6] also used a four-point scale. The gait assessment was performed when the goats walked individually away from the farmer inside the pen, while the observer evaluated the animal from outside the pen. Overall, only 1.7% of the goats whose gait was scored appeared to have lameness. The authors report problems with limited visibility of goats being assessed in crowded pens and difficulties with gait evaluation on old wooden floors, which could be one reason why so few cases of lameness were found. Another reason could be that herd size was generally smaller compared to Reference [5], which found an overall prevalence of lameness of 19.2%. However, the literature is not in agreement on the effect of herd size on lameness. While Reference [21] in goats and Reference [44] in cattle both found a significant difference in lameness frequency as a function of herd size, References [5,20] both found no association. In conclusion, the authors theorised that the true prevalence of mild lameness may be higher than what was recorded, while they claimed to have accurately reported severe cases. Additionally, Reference [6] mentioned the need to further evaluate the reliability of a four-point scale for scoring gait in goats and to consider alternative methods.

In their selection of possible welfare indicators for Brazilian meat goats, Reference [37] chose “lameness” from the AWIN protocol for sheep [38] over “severe lameness” from the corresponding protocol for dairy goats. The AWIN protocol for sheep suggests that lameness be assessed using a four-point scale. According to Reference [37], this method might be more applicable to semi-intensive or extensive housing systems, compared to the “severe lameness” indicator developed mainly for intensively housed goats.

In dairy goats, there is no well-developed, established system for evaluating gait [2]. This lack of standardisation makes it difficult to compare lameness assessment results. For on-farm welfare assessment, as discussed above, identifying only severely lame goats may not be sufficient. Increasing sensitivity by adding more points to a numeric rating scale is, of course, accompanied by an increase in the time required to complete the assessment. Four-point scales have been tested on-farm by References [5,6], with both reporting problems related to assessment location and type of soil. These considerations will be difficult to overcome as small ruminants are kept in very different systems, from very intensive to pasture-based [2]. Increasing the sensitivity of gait assessment may be an easier challenge. Reference [45] suggests the use of a newly developed five-point scale instead of a four-point scale for more sensitive assessment. This new scale was proved to be a reliable scoring system under experimental conditions but needs to be tested in a larger on-farm setting. An alternative to the widely used different variations of numerical rating scales is discussed in Reference [46]. The authors discuss advantages and disadvantages of classical numerical rating scales and of modified visual analogue scales in detail. There is probably no single solution for scoring goats’ gait in the context of animal welfare assessment. Newly developed scoring systems should ideally be tested in large farms under different husbandry conditions.

#### 3.2.2. Body Condition Score

Body condition score (BCS) was defined by Reference [2] as “a method for subjectively assessing the nutritional status of livestock based on an estimate of their body fat”. Reference [47] investigated the use of BCS in the assessment of welfare and concluded that BCS can be used as a routine indicator of welfare. Currently, various scoring systems are used for research or monitoring animals on-farm.

In their protocol to assess dairy goat welfare, Reference [18] chose a three-point visual scoring system with the categories “very thin”, “normal”, and “fat”. This scale was developed by Reference [24] and validated by comparing the scores against results that used a common six-point scale for dairy goats presented by Reference [48]. The observer scored the individual animals within the pen from behind. The simplified three-point scale can be used by inexperienced observers without a significant reduction in reliability and no palpation of animals is necessary [24]. However, this may be limited to Saanen, Alpine and Saanen-Alpine crossbred goats as the scale was not validated for other breeds. Reference [5] assessed goats as “obviously thin”, “normal”, or “obviously fat” by palpating the animals. This method requires restraining of the animals and is therefore more time-consuming than a scoring system using only visual assessment.

When testing their AWIN prototype protocols, References [20,21] both found good on-farm feasibility for BCS. Comparing their results with those of Reference [5], the authors who studied the AWIN protocol generally reported higher scores for both “very thin” and “fat” goats. These differences can largely be explained by different feeding strategies, specifically by the ratio of roughage to concentrate [20,21]. The influence of feeding on BCS was also reported by Reference [6], who found a significant association between higher BCS and feeding automatics when evaluating Norwegian dairy goats.

A modified five-point scale requiring palpation was used by Reference [6] to assess BCS on Norwegian dairy goat farms. The authors found a mean BCS of 2.7 with values ranging from 2.0 to 4.0. Additionally, higher BCS scores were significantly correlated with farmer participation in animal welfare courses. The respective study authors [6] theorise that feeding and nutrition may be a common topic in these courses. 

Reference [47] provides results showing long-term intra-observer-reliability for BCS as an animal welfare indicator in the monthly evaluation of goats over five months. Regarding inter-observer-reliability, Reference [27] found good overall agreement and Reference [24] found substantial inter-observer reliability.

Temporal consistency of the BCS was evaluated by Reference [26], who found that BCS was the only indicator on individual animal level in their protocol that differed significantly between two visits. However, low temporal consistency of BCS can be accepted if variability is caused by seasonal changes, lactation stage, or feeding, as suggested by the authors. Reference [47] also found variations in BCS of goats during a five-month period influenced by different factors than those described by Reference [26].

Therefore, several methods are used to assess BCS in the context of goat welfare, all of which have their advantages and their disadvantages. A visual scoring method provides a simple and valid method that can be used by inexperienced observers and is less stressful for the goats as no palpation is required. Concurrently, the authors clearly indicate that their approach is intended as a quick initial assessment and should not replace more traditional five- or six-point scales for BCS. A combination of these methods could be ideal for obtaining a quick overview of the body condition of a herd and then performing more detailed assessments of individual animals. Further work, specifically on goats, is needed because methods for other small ruminants such as sheep do not translate very well to goats due to anatomical differences in fat distribution [47].

#### 3.2.3. Qualitative Behaviour Assessment

Qualitative behaviour assessment (QBA) is used as a “whole-animal” approach to evaluate the quality of animal behaviour using a list of descriptors such as “content” or “fearful” [49]. In their review of animal-based indicators of dairy goat welfare, Reference [2] reports that QBA has been tested and validated in many other animal species, but they found no similar work for goats. 

An initial approach to assess QBA at a group level was conducted in Reference [6], which adapted descriptors from the Welfare Quality^®^ protocol for dairy cows resulting in five terms used for the assessment. These terms were “fearful”, “calm and indifferent”, “inquisitive/interested”, “aggressive”, and “resting”. Observers rated groups of goats using visual analogue scales for 20 min. The authors found negative associations between health measures (ear tears, skin lesions) and the descriptor “calm and indifferent”. In summary, Reference [6] conducted efficient assessment of a few behavioural expressions in dairy goats.

Reference [2] selected QBA for the prototype AWIN protocol for dairy goats. In the following, References [20,21] both used a list of 13 descriptors for goat emotional state and found good on-farm feasibility. Both studies found that farm size did not have an effect on QBA scores. A limitation mentioned by Reference [21] is that observers were only moderately trained and that using the QBA requires thorough practice to standardise the assessment.

Using results from the AWIN prototype test in a total of 60 goat farms, Reference [25] searched for possible relations between QBA and quantitative animal- or resource-based measures. The authors found few significant relations. Good hair coat condition was associated with goats that were more “relaxed” and “content”. Work in other animal species also considered QBA’s relation to physiological measures [50]. Reference [51] did not find many relations between QBA and animal-based indicators either, which supports the finding of Reference [25]. The authors consider QBA as an important complementary tool alongside quantitative animal-based indicators and point to its potential use for discussing animal welfare issues with farmers. 

Reference [52] tested QBA for dairy goats under different housing conditions in 16 Italian farms, where the goats had additional access to pasture in eight of these farms. Two observers independently scored groups of animals on a list of 16 behavioural descriptors. The assessors used visual analogue scores to qualitatively assess the descriptors. The authors found that access to pasture promotes complex natural behavioural patterns in goats, which may positively influence QBA scores. Additionally, goats kept indoors (no access to pasture) showed higher scores for negative moods such as being aggressive or irritated. However, no clear separation could be made depending on husbandry system, as some farms with indoor housing also achieved very positive QBA results. Management factors such as stocking density and environmental factors such as time of the day could also have influenced the scores and need to be taken into account when assessing animal behaviour according to Reference [52]. The results of this study were used to modify the list of descriptors used in the AWIN protocol for goats [18]. 

Reference [37] discusses modifying the QBA from the AWIN protocol to make the indicator more suitable for meat goats. In their literature review on positive welfare indicators in ruminants, Reference [53] also points out the usefulness of QBA to highlight positive emotional states in goats.

In terms of reliability, Reference [52] assessed inter-observer reliability of the QBA and found good agreement on the ranking among farms and for some individual behavioural terms within farms, while agreement, however, was generally low for individual behavioural terms within farms. To some extent, the authors explain the discrepancies by the different professional backgrounds of the observers, one being a veterinarian and the other an animal scientist. Reference [52] suggests more practice with visual analogue scales and especially more on-farm training to improve inter-observer reliability scores. Evidence in other animal species such as dairy cows [54] also suggested good inter-observer agreement. 

#### 3.2.4. Human–Animal Relationship

A good human–animal relationship (HAR) is one of the 12 criteria included in the European Welfare Quality^®^ program [19]. In their review of HAR in farm animals (ruminants, pigs, poultry, fur animals, and horses), Reference [55] defines a positive relationship between farm animals and humans as low levels of anxiety in animals or animals having high levels of trust in humans. Examples of actual ways to test HAR in goats are avoidance distance tests [56] or “handling” tests [6]. Reference [55] also points out possible benefits of good HAR, such as reducing stress in animals, and noted that a positive relationship between animals and humans required regular, intense, and long-term human contact. In their review of goat welfare indicators published in 2014, Reference [2] stated that this topic has rarely been studied in goats. However, since then, different types of tests have been developed and tested to obtain information on the level of fear or confidence of goats towards humans. 

Reference [56] tested an established, bovine-specific avoidance distance test on cows and goats. The avoidance distance (AD) was defined by the authors as the distance at which the goat showed first signs of an avoidance reaction. Reference [56] found that AD tests are valid for assessing the welfare of goats on farms and are usually easy to perform.

The avoidance distance test with modifications according to Reference [56] was included in the prototype of the AWIN protocol. Both studies agreed that AD results depend on farm size. However, while References [20,56] found better HAR in smaller farms, Reference [21] found statistically higher scores for two aspects of the test in larger farms. Reference [21] notes that feasibility and results of AD tests may also depend on breed, farm size, and production system. In addition, Reference [26] found conflicting results when assessing the consistency of AD tests over time. All these limitations led to the exclusion of the avoidance distance test from the final AWIN protocol [7].

Another report on problems with AD assessment came from Reference [57], which tested three HAR-tests including AD on 12 dairy goat farms. With the help of technical advisors, the researchers classified farms as having “good” or “poor” HAR (judged by expert assessment) and found that their results from AD testing correlated with this classification. Therefore, high validity of this indicator for assessing HAR was assumed. However, the authors stated that AD tests can be time-consuming, depending on the scale of the tests, and that training is required for correct assessment. In addition, pen size could influence the goats’ behaviour towards humans. Reference [57] also mentions the presence of bucks in the pen interfering with AD assessment. If present, bucks would approach the observers first, while females would not be able to make contact. Reference [6] faced similar problems to References [56,57] in that they encountered significant limitations in feasibility when testing AD on farms. The authors reported strong avoidance behaviour by many goats on some farms, but also goats congregating closely around the observer on other farms, which prevents standardised data collection.

Other tests for HAR conducted by Referencce [6] are a “handling test” and a “chin contact test”. On most farms, farmers showed neutral (neither positive nor negative) interactions with the goats in the handling test. A limitation of the handling test noted by Reference [6] is that livestock farmers may introduce some bias when they know their behaviour is being observed. Positive effects of farmer’s positive actions towards their goats were shown by Reference [58]. In their work, stress was lower in petted goats, and there may have been a positive effect on overall health from petting. The results from Reference [58] need to be interpreted carefully as the sample size of goats was quite small (n = 16). In relation to a farm with quite high levels of fear in goats, Reference [6] theorises that in order to handle more fearful goats, farmers are likely to have to chase them more, which further increases their fear response.

Validity and feasibility of a voluntary approach test were tested by Reference [57]. The authors found that this type of HAR test with a stationary observer is more suitable for goats than tests with moving observers. This is because goats are curious animals that rarely have interactions with humans inside the pen. The authors recommend the inclusion of the test in animal welfare assessment protocols and emphasise the importance of standardised tests. As with the avoidance distance, Reference [57] recommends not testing in pens with bucks inside.

The latency to the first contact as described by Reference [57] was tested in the prototype AWIN protocol [21]. Contrary to expectations, latency to first contact was lower in large farms, indicating better HAR. The authors state that their results could be due to differences in breed distribution on farms of different sizes. Smaller farms generally had a greater diversity of breeds including some that are considered more suspicious of humans. This is in line with the findings of Reference [59]. Nevertheless, it is most likely not possible to make general statements about the relation between farm size and HAR, especially given the previously discussed contradictory results for AD.

A set of four HAR tests was investigated for validity and feasibility by Reference [60]. The authors recommended two of their tests that reflect the HAR of goats and that showed good on-farm feasibility. The first test consisted of a person walking parallel to the feed barrier. Reference [60] mentions that the distance to the feed barrier still needed adjustment as there was limited space on some farms. HAR assessments with a moving test subject are particularly difficult to perform because factors such as the speed of movement and the posture of the human can influence the results [55]. The second test proposed by the authors is an avoidance test like the one described and tested by Reference [56] but with a focus on groups of animals rather than individuals. This is because during the initial testing, the authors encountered problems where goats showed group behaviour, which led to a bias in the individual animal score. This agrees with Reference [6], which encountered similar problems when conducting HAR tests. Testing at the group-level, in particular the proportion of goats accepting touch, is thought to represent the goat’s level of confidence in humans. The authors also mentioned various influencing factors, such as the presence of horns [61] or negative interactions between goats and milkers which they believe affect HAR. Another factor that should be considered when assessing HAR is the production system. Reference [59] found that animals and keepers in intensive dairy goat farms had better results for latency to the first contact test compared to semi-intensive farms. Their results are explained by a higher contact rate and the fact that goats in intensive production systems are more accustomed to contact with different people. 

Goats sneezing as a possible indicator of HAR was suggested by farmers [2] and tested by Reference [57]. Sneezes were defined according to Reference [62] as alarm sounds produced by goats to warn their conspecifics. The authors reported a high feasibility of the indicator but recorded a very low occurrence of sneezes. Therefore, the use of sneezing to measure HAR in an agricultural setting could not be supported [57].

Human–animal relationship testing appears to be a young but promising field in goat welfare research, as several publications in recent years have discussed different tests and their relevance for the field of welfare assessment. The variety of factors influencing the results, e.g., housing or stockpeople’s behaviour create a challenging environment for future work. HAR tests would most likely benefit from standardisation to minimise observer bias and to generate more comparable data on dairy goats.

#### 3.2.5. Skin Lesions and Hair Coat Condition

Lesions on goats’ skin was named as a possible indicator of poor health by Reference [2]. Skin lesions were identified as prevalent health issues in dairy goats by References [5,6]. Comparison of the prevalence found in different studies is difficult, as definitions of skin lesions and their scoring systems show high variation. While Reference [6] categorises these lesions as either “mild or severe”, References [5,20] define skin lesions as skin damage or hair loss and further distinguish them according to location of occurrence. The latter two studies chose different locations (e.g., body, neck, head, lower legs) in their respective scoring methods, and Reference [5] even included ear tears. Reference [5] suggests different causes for the lesions depending on the location. Body and neck lesions, mainly consisting of hair loss, suggested environmental reasons such as bars at the feeding place or trauma, while lower leg lesions were possibly indicative of ectoparasitic infections.

Skin lesions were not included within the final AWIN protocol because of low inter-observer reliability presented by Reference [27]. Manual restriction for assessment of lesions might be beneficial as reliability was generally higher when goats were manually restrained rather than at the feeding rack. Neither of References [5,6], which both tested this indicator in dairy goat farms, reported any data on reliability. 

In literature, knee calluses are often listed as a separate welfare indicator but are briefly discussed here in the context of skin lesions. Reference [5] describes this indicator as alterations in the carpal areas of goats’ front limbs including hair loss, scabbing, skin damage, and callus formation. The same authors state that mild knee callus may likely be a normal anatomical feature in goats and that more research is needed to investigate at what stage these alterations become welfare issues. AWIN researchers tested out knee calluses in their prototype protocol and reported low on-farm feasibility [7].

Farmers and veterinarians regularly use goats’ hair coat condition to monitor the animal’s health status [22]. According to Reference [2], hair coat condition is a promising indicator to reflect the welfare criteria of “absence of injuries” and “absence of disease”. The validity and reliability of this indicator in dairy goat welfare assessment were investigated by Reference [22]. In a total population of 1300 Saanen breeding goats in Portugal and Italy, animals with a total or partial rough hair coat were identified visually. A rough hair coat was defined as being either shaggy, matted, rough, scurfy, or having hair longer than normal. Animal dirtiness was not counted as a rough hair coat. Animals with complete fur cover; an even coat; and shiny, glossy, sheen, adherent, and homogeneous hair were characterised as having a normal hair coat. When comparing two sample groups, the authors found goats with a rough hair coat to be in poorer nutritional condition and health status compared to goats with a normal hair coat. Furthermore, Reference [22] shows this indicator to be feasible on-farm and to have high inter-observer reliability. The high mean prevalence of a rough hair coat condition of 15.7% (Portugal) and 25.5% (Italy) recorded in this study demonstrates its importance in welfare assessment. Similar prevalence of 24.1% [20] and 22.9% [21] further point out hair coat condition to be a major welfare issue in dairy goats in Portugal and Italy, respectively.

Consistency over time of hair coat condition was tested by Reference [26], which found this indicator to be sensitive to alterations on an animal basis and on a management basis, resulting in a lack of consistency over time. Nonetheless, the indicator was included in the final AWIN protocol in which an assessor identifies goats with a rough hair coat from outside the pen [7].

To conclude, both skin lesions and hair coat condition can be valid indicators of goat welfare. Issues in reliability and consistency over time should be addressed by further on-farm testing and standardising scoring techniques. These two indicators could also be used in semi-intensive or extensive systems [37,59].

#### 3.2.6. Claw Overgrowth

Overgrown claws are clearly a major welfare issue in intensive dairy goat farms with prevalence ranging from 35.5% [21] to 79.8% [5] of animals affected. Overgrown claws in goats are associated with deformation [63] and lameness [5,41,63]. Scoring methods to evaluate goats’ claws are numerous and differ in assessment techniques and scales used to score the claws. Reference [6] lifted feet to score the claws on a four-point scale (mild, severe, extreme, deformed). Moderate and severe overgrowth was differentiated by Reference [5]. The AWIN protocol [18] assessed claws as being either normal/moderately overgrown (acceptable) or as being overgrown (unacceptable). For this visual binary scoring method, only rear claws were evaluated. 

A subjective visual assessment using photographs of goat claws was validated against objective measurements by Reference [64]. The authors suggested using three-point scales (normal, moderate, and severe overgrowth) to score toe length, heel shape, claw shape, and claw splay, and a binary scale to score fetlock shape. Their method showed high levels of accuracy and reliability but required extensive training prior to assessment. According to Reference [64], the feasibility of their method on-farm with live animals still needs to be tested. 

Reasons for overgrown claws include housing on straw bedding (lack of claw wear) [5,20], inadequate foot trimming [5,43], and no or little access to natural pasture [65]. Larger dairy goat farms were shown to have a higher prevalence of overgrown claws [20,21]. In order for accuracy and feasibility of goat claw scoring applied on-farm to be increased, a visual binary assessment appears to be the most appropriate method to date [7,64].

#### 3.2.7. Cleanliness

There is little information available on the effect of dirtiness on goat welfare. In dairy cattle, Reference [66] shows that udder and leg hygiene scores are associated and that there is a significant correlation between udder hygiene score and the prevalence of intramammary infection. These results may also apply to dairy goats [5], but specific investigations will be necessary as goats are generally much cleaner than cattle due to drier faecal matter and straw bedding being more common [2,5]. Cleanliness of dairy goats was evaluated by AWIN researchers [20,21] who visually recorded any muddy or wet areas and yellowish hair as goats being dirty. The authors concluded that visual assessment of dirtiness in goats depends on coat colour and therefore on breed, making this method unsuitable for the final version of the AWIN protocol. The AWIN researchers consequently replaced this indicator with the visual assessment of quantity and cleanliness of straw bedding inside the pen. Other authors who assessed goats’ cleanliness did not report the specific problem of coat colour complicating the evaluation of animals’ dirtiness [5,6,59]. One reason might be that the AWIN protocol heavily focuses on on-farm feasibility and fast assessment, leaving assessors with little time to inspect individual animals. Assessors applying other protocols than AWIN might have spent more time on this indicator, thus being able to correctly assess cleanliness independent of the animal’s coat colour. However, drawing comparisons between the different above-mentioned methods is difficult as they vary greatly in definition of dirtiness and scoring system. Of course, animal cleanliness is also influenced by housing and management factors such as moving animals around, cleanliness of walkways, or access to pasture/outdoor areas [5].

Faecal soiling is considered a separate welfare indicator by some authors and grouped under the umbrella term cleanliness by others. Reference [7] defines this term as “the presence of manure below the tail head” and use a visual assessment of this indicator to detect diarrhoea. In general, faecal soiling can indicate problems in nutrition and digestion in dairy cattle [67]. To conclude, assessment of goats’ cleanliness may require a some time in order to correctly score animals considering possible bias introduced by different coat colours. 

#### 3.2.8. Udder and Teat Abnormalities

Lesions of udders and teats as well as alterations in udder conformation can affect dairy goats’ welfare and production value [21,68,69,70]. A great variety of specific udder and teat indicators have been used in the assessment of dairy goat welfare. Different udder and teat abnormalities include, for example, skin lesions, missing glands, accessory teats, pendulous udders, inflamed skin, swellings, asymmetry between the glands, and clinical mastitis [5,6]. 

It is important to note that assessment technique for udder and teat abnormalities greatly influences which aspects can and which cannot be evaluated on-farm. In contrast to Reference [5], which only visually assessed udders and teats, Reference [6] also palpated the animals’ udders. Additional palpation can help to identify pathologies in udders that appear clinically normal at visual examination [71]. A thorough examination of udder and teats including palpation may be very insightful for welfare assessment but of course is quite time-consuming. This is probably one of the reasons why AWIN researchers chose only one indicator (udder asymmetry) to visually evaluate udder health in their protocol. Udder asymmetry is the most prevalent abnormality of the udder in dairy goats [5,6]. Asymmetry of the udder in dairy goats is a chronic change [21] and is associated with intramammary infection [68]. An asymmetric udder is defined as one half being at least 25% longer than the other, not including the teats [7]. Udder asymmetry can be assessed on goats restrained at the feeding rack [7] or as suggested by References [2,5], in the milking parlour. A common scoring system for udder and teat abnormalities could provide a basis for data comparisons [20]. Another goal for future research might be to find the most valid variables out of the ones proposed in existing protocols in order to perform udder and teat health assessment in a reasonable time frame.

#### 3.2.9. External Abscesses

Abscesses are defined by Reference [72] as swollen areas in the body tissue with purulent content, due to bacterial infections. *Corynebacterium pseudotuberculosis* is the most common pathogen leading to abscess formation in small ruminants and may cause caseous lymphadenitis [73]. While internal abscesses cannot be easily diagnosed on-farm, external abscesses, mostly located in lymph node areas, can be assessed visually with a quick examination of the animal [2]. This indicator was validated in Reference [72]. Their results indicated that external abscesses may have a negative influence on goats’ welfare condition. Occurrence of external abscesses may depend on farm size because early detection of the highly contagious caseous lymphadenitis is more difficult in larger herds [20]. This indicator can be scored with a binary system (presence or absence) where an assessor inspects the head, neck, shoulders, hindquarters, and udder area of goats [7]. External abscesses can quickly be assessed on-farm and could be a useful welfare indicator for goats. As stated in Reference [72], their results were to some degree limited by their rather small sample size of only 35 Saanen dairy goats. A more comprehensive validation of this indicator might be reached by using a larger sample population in future work.

#### 3.2.10. Nasal, Ocular, and Vulvar Discharge

Nasal and ocular discharge in goats are usually a consequence of a pathogenic challenge or inadequate environmental conditions [6,18]. These two indicators have been scored on-farm by several authors for dairy goats. Fairly similar results were found for nasal discharge by References [5,6] and AWIN researchers. Regarding ocular discharge, Reference [6] found a prevalence of 35.6% in their study population, while References [5,20,21] reported 6%, 0.9%, and 9%, respectively. One reason for these differences might be that Reference [6] included any sort of discharge in their assessment, while, for example, Reference [20] used more narrow definitions for their evaluation of nasal and ocular discharge. 

Vulvar discharge was found on less than 1% of goats when AWIN researchers tested their prototype protocol. This low prevalence led to the exclusion of this indicator from the final protocol [7]. In contrast, Reference [5] found 5% of their study population to have vulvar discharge. This difference is likely because AWIN researchers did not include haemorrhagic discharge, while Reference [5] recorded any type of discharge and stated that in 92% of cases in their assessment, the discharge was haemorrhagic. Discharges of different natures have been used as welfare indicators on-farm and appear to be easy to score as only their presence or absence is noted in most existing protocols. However, there is still a need to investigate which types of discharges should be considered for welfare assessment and how environmental factors influence this animal-based indicator.

#### 3.2.11. Thermal Stress

Thermal conditions for dairy goats housed indoors include an optimal temperature of 10–18 °C, relative humidity between 60 and 80%, and wind speed of 0.5 m/s [74]. Outside of these ranges, goats may experience varying degrees of thermal stress that negatively affect goat welfare [75,76]. Several environmental or resource-based indicators (e.g., temperature, relative humidity) have been identified and successfully used in goat welfare science [75]. However, animal-based indicators more accurately reflect on goats’ welfare status because this method can account for characteristics such as breed [77] or physiological adaptations to weather conditions [23,47]. Animal-based indicators of thermal stress in dairy goats have been validated in Reference [23]. The authors examined lactating Alpine and Saanen goats from January to July at different times of the day and measured environmental conditions. Hair horripilation (score 1) and shivering (score 2) were used to identify cold stress, while elevated respiration (score 1) and panting (score 2) were used to recognise goats with heat stress. These two sets of descriptors can be used to visually score dairy goats on-farm, resulting in valid information on welfare. As compared to other suggested indicators such as skin temperature [76] or BCS [47], the assessment method presented by Reference [23] can be performed quickly and does not require any additional tools. The authors even propose collapsing their scoring system to a binary assessment (presence or absence of heat-/cold stress) in order to further increase feasibility. This suggestion was adopted by AWIN researchers when they chose thermal stress for their assessment protocol [7]. Training of assessors is essential for this indicator because confounders such as underlying health issues may also cause thermal stress behaviour such as elevated respiration which has to be differentiated from actual heat or cold stress [23]. A visual assessment of thermal stress in dairy goats using specific descriptors can validly reflect on their welfare. Assessment of this indicator takes little time but may be complicated by confounding factors such as underlying health issues which could reduce the feasibility. The use of this indicator for meat or fibre goats or goats kept more extensively is subject to future research. Existing scores such as the one described by Reference [23] can be used and modified to create assessment methods for other goat breeds in different production systems [37].

#### 3.2.12. Other Indicators Used for the Assessment of Welfare in Goats

Some indicators are employed in different welfare assessment schemes for goats but lack sufficient research in the literature to be discussed in detail. We briefly discuss these indicators here.

Kneeling (goats dropping on their front knees/carpal joints [5]) can have different meanings depending on the location in which goats show this behaviour [20]. Goats kneeling at the feeding rack is assumed to be a coping strategy for errors in facilities design while kneeling inside the pen is most likely due to pain in the locomotor system [5].

In intensive and semi-intensive dairy goat farms, there is often a competitive environment when it comes to feed and water intake [2,78,79], especially if the stocking density is high [21]. Queuing is usually adopted by goats as a coping strategy if there is competition for feed or at the drinker [2,79]. Queuing behaviour in dairy goats can serve as an indicator for limited access to feed and a negative emotional state, both compromising animal welfare [2].

According to anecdotal evidence from farmers and technicians, obviously sick or dull goats often isolate themselves from the group, stand immobile, and face housing structures such as walls [2]. This behavioural phenotype is also described as “oblivion” and may be helpful to recognise animals in poor health [62].

Frequent agonistic interactions (such as head-butts) between goats can cause injuries and become a health and welfare issue [61]. It is suggested by Reference [5] that partial horns (improperly disbudded horns) may be more damaging than full horns for other goats in the group as well as for the animal carrying them. There is still a need to further investigate the use of specifically partial horns as an indicator of welfare in goats.

Lying behaviour in goats has been studied with the use of devices such as acceleration loggers [80] or video recorders [81]. However, these methods would not be feasible for animal-based on-farm welfare assessment. Instead, AWIN researchers chose to assess the number of goats with abnormal lying postures at a certain moment for their prototype protocol. The nature of abnormal lying in dairy goats is not well understood, but Reference [20] theorises that respiration difficulties, attempts to dissipate heat, lesions to the sternum, or pain in the front legs might play a role. The authors did not further develop this indicator as it was recorded with a very low prevalence (<1%) when testing their prototype protocol.

Amongst other things, swellings of lymph nodes and joints have been proposed by Reference [6] to cover “freedom from pain, injury or disease” from the five freedoms principle of animal welfare [82]. The same indicators have also been applied by Reference [5] in on-farm welfare assessment of goats.

The FAMACHA^©^ method is used to identify animals in need of anthelmintic treatment by comparing their mucous membranes with a colour chart. This principle is based on the presence of *Haemonchus contortus*, a gastrointestinal nematode that can cause anaemia in its host [83]. This system appears to work best under resource-poor conditions where goats can quickly become infested with a high burden of *H. contortus* [84]. Despite this fact, the FAMACHA^©^ method can be a valuable tool in Europe as well if a herd is harbouring resistant *H. contortus* [83]. This indicator has not been used in any welfare assessment protocols yet.

More possible indicators for the assessment of goat welfare are listed in Table 2. These indicators are only mentioned sporadically in the literature and/or have not been validated and tested in the context of on-farm welfare assessment. Some of the aforementioned indicators are included in established welfare protocols, such as queuing and oblivion in the AWIN protocol. Such inclusion likely occurred on request of stakeholders or on the basis of anecdotal evidence. Although the indicators appear to work well within the framework of the respective protocols, these indicators require further scientific investigation in the future.

## 4. Conclusions

The aim of this review was to provide an overview of the current literature on welfare assessment for goats by identifying reported animal-based indicators. Among the literature, we found several protocols which include a fixed list of multiple indicators which have been tested by the respective creators on-farm. One of these assessment schemes, the AWIN protocol for adult dairy goats contains indicators that are mostly validated, highly feasible for on-farm usage, and generally reliable and consistent. Other protocols, such as the welfare assessment of Norwegian or U.K. dairy goats [5,6], do not provide this degree of scrutiny of the indicators. However, these protocols still provide valuable information on indicators not included by AWIN researchers and present an approach that is not as limited by high feasibility and farmers acceptance as the AWIN protocol.

Regarding individual indicators, only a few have been thoroughly studied and validated specifically for their on-farm use to assess welfare in goats. Namely, lameness, BCS, QBA, and human–animal relationship tests are indicators which are widely used and discussed in various studies. The usage of other indicators for welfare assessment, such as hair coat condition and thermal stress, appears to be covered by only few scientific studies thus far. This indicates that indicators are sometimes selected on the basis of anecdotal evidence alone. Although experience likely also provides valid and working indicators, the development of objective, reliable, and (ideally) universal indicators requires evaluation and validation through the scientific process.

Researchers applying the above-mentioned indicators in on-farm assessments mostly have the advantage of choosing between different existing scoring systems depending on their aims regarding accuracy, reliability, and feasibility. For many other promising indicators such as oblivion or improper disbudding, future research on their validity and on-farm use would be beneficial.

Most of the literature addresses the welfare of dairy goats in intensive production systems; however, some indicators might be applicable for bucks, kids, meat goats, or goats in extensive production systems as well. The variety in these production systems, from goats kept outdoors and milked manually to goats inside farms with milking parlours, requires the development or modification of indicators according to the specific attributes of these different production systems. Specifically, the degree of mechanisation in farm work and the amount of human handling goats receive might have an impact on goat welfare and is subject to future research. However, in a scientific context, indicators are usually developed and validated under strict homogenisation of influencing factors in order to limit the number of parameters to consider and, hence, reduce the necessary sample sizes. In this respect, it may be advantageous for validation studies to use a heterogeneous collection of testing farms, such that subsequent meta-analyses may extract the influences of influencing factors.

Another important focus of future work is to find indicators of positive welfare in goats, such as, for example, vocalisations [90]. In recent years, more work has been directed towards the identification of positive welfare indicators [3], yet an implementation of such indicators into a working protocol is still missing. A particular challenge appears to be the definition of positive welfare, as an absence of negative experiences does not necessarily translate to a positive emotional state [53].

Advantages and disadvantages of indicators are highlighted in this review and are discussed in a broad sense without too much emphasis on single aspects such as feasibility. With the help of farmers, experts, stakeholders, and consumers, a system can be developed to objectively quantify animal welfare in goat farms on the basis of indicators presented here. Such a protocol could be used in regional or national goat populations with the long-term goals of improving the animals’ welfare status and investigating the impact and success of implemented health and welfare programs. Since the submission of this manuscript, two further studies had been published regarding general goat welfare indicators with focus on the Americas (USA, Mexico) [8,9]. For their methods, both articles again draw together “the available literature on goat welfare assessment (...) including assessment protocols that had been used previously (...).” [8]. This highlights the importance of a collection of the available information on goat welfare indicators, which can be used to facilitate this step in the future.

## Figures and Tables

**Table 1:** List of goat welfare indicators included in each of the presented assessment protocols. Indicators included in a protocol are marked by X. For the AWIN protocol and the Anzuino protocol., the inclusion in the group level and individual level is stated. For the Leite protocol, animal-based and resource-based indicators are listed separately. Resource-based indicators are listed for completeness but are not discussed in detail. Similar indicators, such as udder asymmetry, udder abnormalities, or udder pathologies are summarised as single row, although they may be evaluated as separate indicators within a protocol.

Indicator	AWIN Protocol		Anzuino Protocol		Muri Protocol	Leite Protocol	
	Group	Individual	Group	Individual		Animal-Based	Resource-Based
Lameness	X			X	X	X	
Body condition score		X		X	X	X	
Abscesses/swellings		X		X	X	X	
Cleanliness/fecal soiling		X		X	X	X	
Discharches		X		X	X	X	
Qualitative behavioural assessment	X				X	X	
Human-animal relationship	X				X	X	
Overgrown claws		X		X	X		
Udder abnormalities/asymmetry/pathologies		X		X	X		
Lesions				X	X	X	
Improper disbudding	X			X			
Kneeling	X		X				
Queuing	X					X	
Hair coat condition	X					X	
Oblivion	X					X	
Thermal stress	X					X	
Bedding	X						
Oral behaviour			X				
Dyspnoe			X				
Coughing			X				
Pruritus							
Obviously sick/dull			X				
Chest girth					X		
Pinna pathologies					X		
Leg injuries						X	
Water availability							X
Access to shade and shelter							X
Stocking density							X
Cleanliness of facilities							X

**Table 2:** Potential welfare indicators for goats that lack on-farm validation and/or have little literature available.

Indicator	Reference
Abnormal oral behaviour	[2,5]
Chest girth	[6]
Dyspnoe, respiration rate (variability)	[5,85]
Exploratory behaviour	[2,53]
Heart rate (variability)	[85,86,87]
Ill-thrift/weight loss	[47,88,89]
Latency to display behaviour post-partum (goat kids)	[53]
Mortality, condemnation rates	[65,88,89]
Presence of ectoparasites	[6]
Pruritus	[2,5]
Stereotypies	[59]
Synchronisation of lying	[53]
Time spent lying (by a wall)	[53]
Vocalisation	[2,59,85]

## Data Availability

No new data were created or analyzed in this study. Data sharing is not applicable to this article.
